# A novel external beam radiotherapy method for cervical cancer patients using virtual straight or bending boost areas; an in-silico feasibility study

**DOI:** 10.1186/s13014-021-01838-x

**Published:** 2021-06-14

**Authors:** Luca Cozzi, Sushil Beriwal, Esa Kuusela, Supriya Chopra, Hester Burger, Nanette Joubert, Antonella Fogliata, Jai Prakash Agarwal, Pat Kupelian

**Affiliations:** 1 Radiotherapy and Radiosurgery Department, Humanitas Research Hospital and Cancer Center, Via Manzoni 56, 20089 Milan-Rozzano, Italy; 2 Department of Radiation Oncology, UPMC Hillman Cancer Center, University of Pittsburgh School of Medicine, Pittsburgh, USA; 3 Department of Radiation Oncology, Advanced Centre for Treatment Research and Education in Cancer, Tata Memorial Centre, Homi Bhaba National Institute, Mumbai, India; 4 Varian Medical Systems, Palo Alto, USA; 5 Radiation Oncology Dept, University of California, Los Angeles, USA; 6 Varian Medical Systems Finland, Helsinki, Finland; 7 Division of Medical Physics, University of Cape Town and Groote Schuur Hospital, Cape Town, South Africa

**Keywords:** VMAT, RapidArc, Cervix uteri cancer, Spatially fractionated radiation therapy, Virtual brachytherapy

## Abstract

**Aim:**

To investigate the potential role of a novel spatially fractionated radiation therapy (SFRT) method where heterogeneous dose patterns are created in target areas with virtual rods, straight or curving, of variable position, diameter, separation and alignment personalised to a patient’s anatomy. The images chosen for this study were CT scans acquired for the external beam part of radiotherapy.

**Methods:**

Ten patients with locally advanced cervical cancer were retrospectively investigated with SFRT. The dose prescription was 30 Gy in 5 fractions to 90% target volume coverage. Peak-and-valley (SFRT_1) and peak-only (SFRT_2) strategies were applied to generate the heterogeneous dose distributions. The planning objectives for the target (CTV) were D_90%_ ≥ 30 Gy, V_45Gy_ ≥ 50–55% and V_60Gy_ ≥ 30%. The planning objectives for the organs at risk (OAR) were: D_2cm3_ ≤ 23.75 Gy, 17.0 Gy, 19.5 Gy, 17.0 Gy for the bladder, rectum, sigmoid and bowel, respectively. The plan comparison was performed employing the quantitative analysis of the dose-volume histograms.

**Results:**

The D_2cm3_ was 22.4 ± 2.0 (22.6 ± 2.1) and 13.9 ± 2.9 (13.2 ± 3.0) for the bladder and the rectum for SFRT_1 (SFRT_2). The results for the sigmoid and the bowel were 2.6 ± 3.1 (2.8 ± 3.0) and 9.1 ± 5.9 (9.7 ± 7.3), respectively. The hotspots in the target volume were V_45Gy_ = 43.1 ± 7.5% (56.6 ± 5.6%) and V_60Gy_ = 15.4 ± 5.6% (26.8 ± 6.6%) for SFRT_1 (SFRT_2). To account for potential uncertainties in the positioning, the dose prescription could be escalated to D_90%_ = 33–35 Gy to the CTV without compromising any constraints to the OARs

**Conclusion:**

In this dosimetric study, the proposed novel planning technique for boosting the cervix uteri was associated with high-quality plans, respecting constraints for the organs at risk and approaching the level of dose heterogeneity achieved with routine brachytherapy. Based on a sample of 10 patients, the results are promising and might lead to a phase I clinical trial.

**Supplementary Information:**

The online version contains supplementary material available at 10.1186/s13014-021-01838-x.

## Introduction

At the global level, the incidence and mortality of cervical cancer are high with more impact in low-mid income countries (LMICs) due to lack of screening, vaccination against the human papillomavirus, and overall limited access to care [1]. Concurrent chemo-radiotherapy is the standard of care, with the radiotherapy component consisting of external beam treatments followed by brachytherapy (BT, intracavitary ± interstitial). The utilisation of the BT boost has been established as providing remarkable benefit in terms of disease control [2, 3]. The omission of brachytherapy is also associated with detriment in survival [4].

The application of brachytherapy is dependent on two factors: (1) availability of brachytherapy facilities, radioactive brachytherapy sources, and associated experienced clinical teams, and (2) contra-indications to BT, primarily due to very advanced stages of the disease. If any of these two factors apply, it would be desirable to expand the radiotherapeutic options panel with practical techniques.

The use of advanced techniques based on external beam therapy has been explored in this perspective. Concerning stereotactic body radiotherapy (SBRT), Albuquerque [5] reported a phase II trial for locally advanced cervical cancer in a phase II trial. The outcomes were inferior with SBRT, and the trial was closed due to the severe toxicity profile. The authors suggested that better case selection might be needed, and the boost volume should be better defined. Small tumours in patients unable to receive standard BT were suggested to be best eligible for SBRT.

In the domain of the alternatives to BT, the concept of spatially fractionated radiotherapy (SFRT), lattice or grid therapy techniques have been proposed already in the 1950s [6], and a variety of solutions have been utilised, requiring either dedicated metallic grids (physical compensators) or multileaf collimators (MLC) [7].

Griffin [8] comprehensively reviewed the state of the art for SFRT, concluding that SFRT technology developments are needed, mechanisms of action should be investigated, and clinical outcomes should be tested in well-designed controlled studies to confirm the relevance and potential of SFRT. Yan [9] outlined the relevance of SFRT in delivering inhomogeneous radiation therapy to various clinical indications such as head and neck, lung breast, gynecologic and sarcoma cases. Zhang [10] suggested that a more standardised approach might facilitate advances in the clinical management of bulky tumours employing SFRT.

Suppose GRID or SFRT based on physical devices might imply some challenges (e.g. the need for dedicated infrastructure). In that case, the virtualisation of the dose patterns generation might simplify many aspects. The removal of additional hardware pieces such as physical compensators, the flexibility of software-based solutions, and the simplified dose calibration issues are the factor of appeals for a MLC-based solution.

Amendola [11] proposed a lattice radiotherapy technique consisting of volumetric modulated arc therapy (VMAT) for gynaecological tumours. Their solution was to distribute manually contoured spheres (15) of 1 cm diameter that were added inside the gross tumour volume and planned to deliver a 2.4 Gy/fraction dose. In contrast, the wider volume received 1.8 Gy/fraction. Pokher [12] proposed an MLC generated lattice pattern of 10 mm diameter and 20 mm centre-to-centre separation. No flexibility in the shape and geometrical features of the patterns were proposed, but the dosimetric results proved the potential appeal of the virtualised frame of work. Choi [13] reported clinical outcomes with virtual or physical GRID devices for palliative radiotherapy in head and neck cancers in a cohort of 21 patients. Murphy, in a retrospective in-silico study, [14] proposed the use of virtual GRID therapy for the treatment of breast cancers in the prone position.

The primary aim of this current in-silico investigation was the creation and validation, at the treatment planning level, of a novel and flexible method enabling SFRT. The scope was to develop appropriate tools to generate some virtual rods of free diameter, length, position and carving. These rods would be used as “seeds” to optimise hot dose regions with a peak-and-valley or peak-only patterns within a given target volume. The key dosimetric objective to be achieved was the simultaneous generation of sufficiently hot dose regions within the target to mimic the clinical benefit of brachytherapy and the minimisation of the organs at risk involvement to reduce the toxicity reported from stereotactic body radiotherapy [5]. The clinical context tested was the boost course in a radiotherapy course for cervical cancer, comparing dosimetric endpoints with reference data from conventional brachytherapy. The idea underlying the SFRT concept presented here is that its use could be extended to any other clinical case suitable for highly heterogeneous dose deliveries.

## Materials and methods

### Patients selection, contouring and dose prescription

This retrospective in-silico investigation was performed on a group of 10 patients selected from an institutional database. The chosen patient data sets were patients with locally advanced cervical cancer (FIGO 2009 Stage IIB). All patients had baseline T2 W MRI images. Soft tissue fusion with contrast-enhanced CT was performed to facilitate delineation of CTV. Patients were chosen such that their clinical target volumes at brachytherapy were representatives of patients with significant residual disease (high-risk CTV > 30 cm^3^) at the time of brachytherapy such that the impact of these relatively large targets could be studied for both target coverage and organ at risk sparing. The target delineation consisted of the high-risk clinical target volume (CTV), including the primary gross tumour volume and the remaining cervix not infiltrated by the tumour as described in [15]. For the planning study purposes, this volume was assumed to be the target for the boost course, either with SBRT or with BT (although in a real clinical case, a new planning CT would be acquired to define it at the end of the first course of radiotherapy). For consistency, it was named CTV. With the assumption described above, for consistency with BT practice and the dosimetric study purpose, no formal expansions were considered to generate a planning target volume (PTV) to be considered to optimise the dose plans. Nevertheless, to appraise the robustness of the planning technique to (minor) setup uncertainties, plans were also appraised for PTVs with margins of 2 or 5 mm from the CTV (PTV2mm and PTV5mm).

The bladder, the rectum, the sigmoid and the bowel bag were considered as Organs at Risk (OAR) for this study.

The dose prescription to the CTV was set to 30 Gy in 5 fractions normalised to the 90% coverage level (i.e. the dose covering 90% of the CTV was normalised to 30 Gy); this was done for consistency with the clinical practice in the case of a brachytherapy boost. Besides the coverage requirement, additional planning aims to the CTV were applied to the volume receiving at least 45 Gy and at least 60 Gy (150% and 200% of the prescription): V_45Gy_ ≥ 50–55% and V_60Gy_ ≥ 30%. These reference values were derived from institutional data from MRTI guided image-based brachytherapy for patients treated as per the GEC_ESTRO guidelines. Cases were chosen randomly, and average V_45Gy_ and V_60Gy_ were computed and compared to those obtained from the dataset used to optimise with the rods.

For the OARs, the clinical aims in the study were defined for the near-to-maximum dose (at 2 cm^3^ of volume). This was set as D_2cm3_ ≤ 23.75 Gy, 17.0 Gy, 19.5 Gy, 17.0 Gy for the bladder, rectum, sigmoid, and bowel, respectively so that near-to-maximum cumulative (i.e. inclusive of phase 1 external beam radiotherapy course of 45 Gy, after conversion to 2 Gy/fraction) would be: D_2cm3_ to the bladder, rectum, sigmoid and small bowel were ≤ 65 Gy, ≤ 80 Gy, ≤ 70 Gy and ≤ 65 Gy respectively. Upper limits to the clinical aims were set to: 19.45, 27.5 21.65, 19.45 Gy, respectively. The planning constraints expressed above are derived from routine BT clinical practice and are tighter than the values reported for SABR by Leung [16].

#### Spatially fractionated structures and dose optimization

**Fig. 1:**
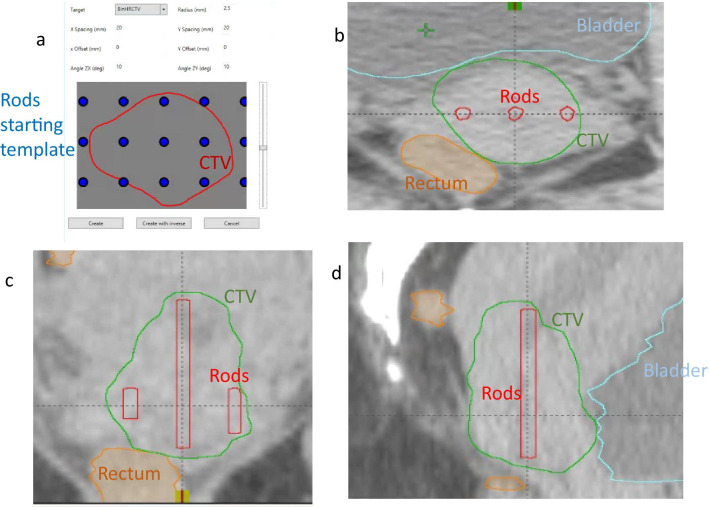
**a** the user interface for the generation of the straight rods in Eclipse; **b**–**d** axial, coronal and sagittal views of the rods with different lengths to further illustrate the various features of the tool; in green the CTV segmentation, in cyan the bladder and in brown the sigmoid/rectum

The primary aim of the in-silico study was to generate highly heterogeneous dose distributions within the target volume. The idea was to distribute the heterogeneity according to a sequence of elementary geometrical virtual patterns (named “rods”) automatically contoured by means of a dedicated script in Eclipse™. Figure 1 illustrates the logic of the procedure. In the first instance, the geometrical 2D grid of rods is defined and positioned within the target volume (panel a). The diameter, spacing, offset of the rod grid, and the rods’ angle (with respect to the DICOM axes in the planning CT) are the free parameters. Panels b-d show the rods (red) within the CTV (green) in axial, coronal and sagittal views. In this version of rod generation, only parallel and straight rods aligned along any angle in the ZX and ZY planes were allowed. An individualised per patient rod setting was defined according to a general template. Three rods per patients were used with one central rod covering approximately the entire length of the CTV; the position of the two shorter lateral rods was defined according to the shape of the CTV and the relative position with respect to the organs at risk.

A second version of the script allowed the generation of “bending rods”. In this case, the logic required the definition of 2 or more seed points (one at the beginning and another at the end of the desired rod extension and optionally one or more intermediate points defining the shape and bending of the rod) within the target volume. A single rod was then generated with cubic spline interpolation from those seeds. The utility of bending rods is better adherence to a patient’s specific anatomy (e.g. the curvature of the uterus or tumour extension). Multiple rods could be generated and eventually joined into a single structure or kept separate. The second generation of the script would enable the assignment of different dose levels to other rods, while in the first version, a single dose level could be assigned to all straight rods. A qualitative assessment of this “bending rods” approach will be presented here in the Additional file 1: complementary materials section.

#### Photon planning

The treatment plans were designed for 6 MV flattening filter free photon beams generated by a Halcyon treatment machine (Varian Medical Systems, Palo Alto, USA) and optimised with the Eclipse treatment planning system v. 16.1 (Varian Medical Systems, Palo Alto, USA) using the photon optimisation engine and the Acuros-XB dose calculation algorithm for the final dose distribution (computed with a cubic grid of 2.5 mm side). The technique chosen for the study was Volumetric Modulated Arc Therapy (RapidArc). Three complete arcs were selected. A separate study not presented in this report demonstrated that the SFRT technique could also be planned on c-arm linacs and does not need to be restricted to a Halcyon system.

Three sets of plans were optimised for each patient: a simple VMAT plan mimicking a stereotactic body radiotherapy approach (SBRT), the SFRT_1 and the SFRT_2 corresponding to the peak-and-valley and peak-only strategies. To realise the peak-and-valley plans, the rods were surrounded by cooler regions (generated starting from a boolean difference between the rods and the CTV with some additional margin) where dose-volume constraints kept doses at 45 Gy or lower. The choice of the peak-to-valley ratio is currently arbitrary and not based on a specific radiobiological rationale. In the present study, rod diameters were set at 5.0 mm, and the centre-to-centre rod separations were set at 15.0 mm.

To obtain heterogeneous dose distributions, the rods (straight or bending) were associated with high dose constraints (in the range of 60 to 80 Gy, i.e. 150–200% of target prescription). In the case of SFRT_1 plans, a minimum dose constraint was applied to the rods while the valley optimisation was obtained using the cooling structure defined above. In the SFRT_2 plans, no negative structure was applied. In both cases, the constraints were also applied to V_45Gy_ and V_60Gy_. The planning quality was evaluated reviewing, also during the optimisation phase, the clinical planning aims reported above.

The risk of underdosing the target volume (in the present study named CTV) in the presence of positioning uncertainties, or possible small anatomical variations, was measured in terms of the expected dose coverage to the virtual PTV expansions with 2 and 5 mm. In addition, two other mitigation strategies were considered: i) all plans were renormalised to PTV2mm (SFRT A) or ii) all plans were re-optimised including PTV2mm and then renormalised to PTV2mm(SFRT B). In both cases, we aimed to calibrate the D_90%_ coverage for the PTV2mm and PTV5mm, setting them to the highest possible value while not violating any of the constraints to the OARs. With this approach, we aimed to demonstrate the feasibility of the proposed technique also when accounting for some setup uncertainties.

### Quantitative assessment of dose-volume metrics

The dose distributions were analysed by means of a set of appropriate dose-volume metrics (corresponding to the planning aims) derived from the Dose Volume histograms (DVH) of the plans. The average DVH for the target and the various OARs was computed with a dose binning of 0.02 Gy to represent the data visually.

To complement the analysis and put the dose plan quality in relation to current practice with standard brachytherapy treatments, two data points for V45 and V60 Gy were extrapolated from an existing database of routine cervical cancer treatment plans from a single institution.

All the data were analyses in parallel as physical doses as well as biologically corrected equivalent doses (in 2 Gy fractions) using the usual relation $EQD2 = ~\frac{{nd\left( {1 + \frac{d}{{\frac{\alpha }{\beta }}}} \right)}}{{1 + \frac{2}{{\frac{\alpha }{\beta }}}}}$ with n = number of fractions, d = dose per fraction, α/β = 3 for the OARs and 10 for the CTV.

To further mimic the case of virtualised brachytherapy, the biologically corrected doses were then summed to an expected-to-be-uniform previous irradiation of 45 Gy from conventional external beam radiotherapy (whatever the technique). This is a simplified approach not considering the possibility of variably sparing the OARs, constituting a worst-case scenario. Dose tolerances for the OARs were updated accordingly.

## Results

### General findings

**Fig. 2:**
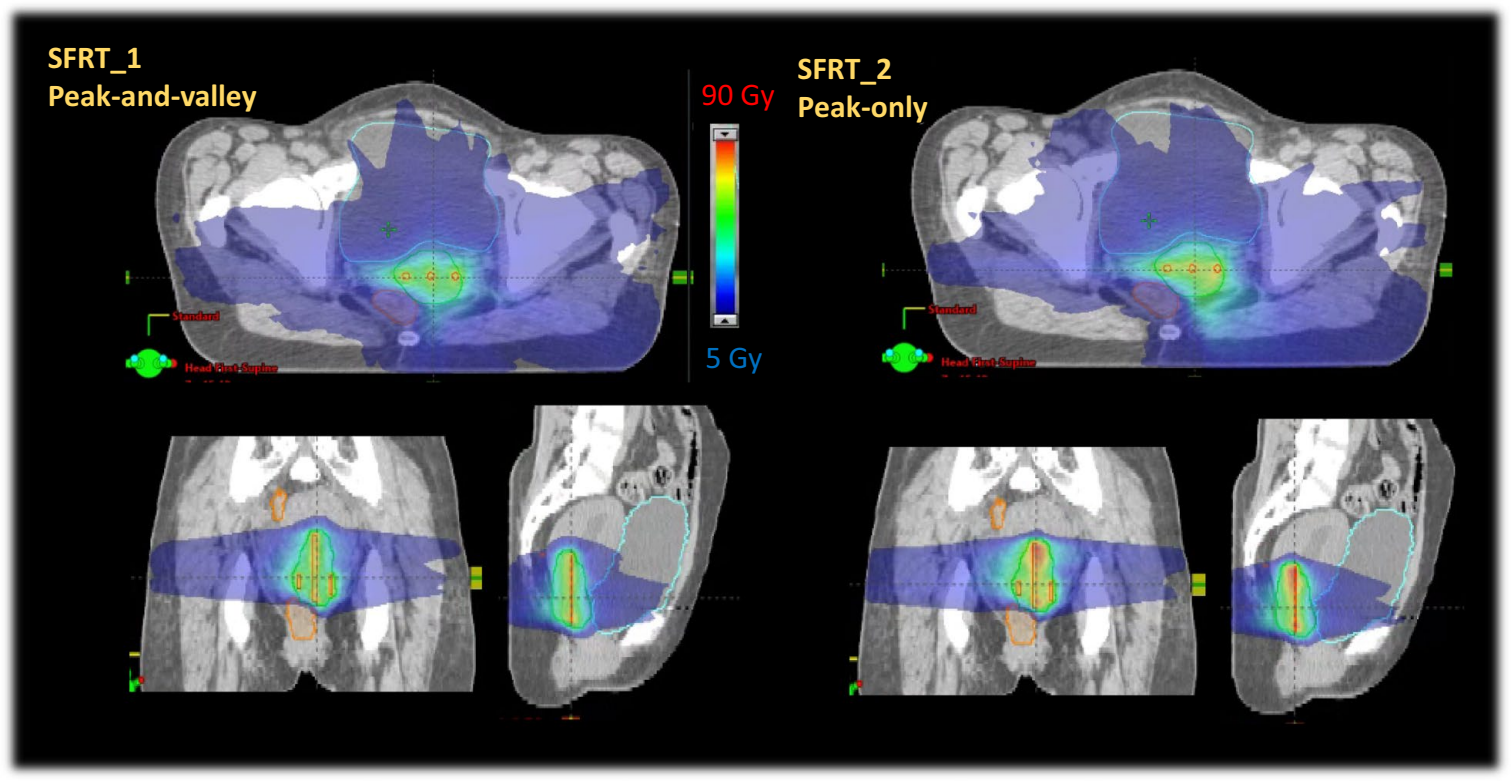
Example of the dose distributions achieved in the CTV for the SFRT_1 and SFRT_2 techniques (peak-and-valley and peak-only). The colour-wash for the dose was set to 5–90 Gy); in green the CTV segmentation, in cyan the bladder and in brown the sigmoid/rectum

The median CTV in this group of patients was 51 cm^3^ (range: 30.1–89.2). For comparison, the median CTV in [5] was 81.7 cm^3^. Figure 2 illustrates at a qualitative level the SFRT dose distributions obtained for the peak-and-valley and peak-only strategies. The dose colour wash is set from 5 to 90 Gy. The figure allows appraising the conformality of the dose to the CTV, the high dose in the volume of the rod and the dose bath characteristic of the external irradiation.

**Fig. 3:**
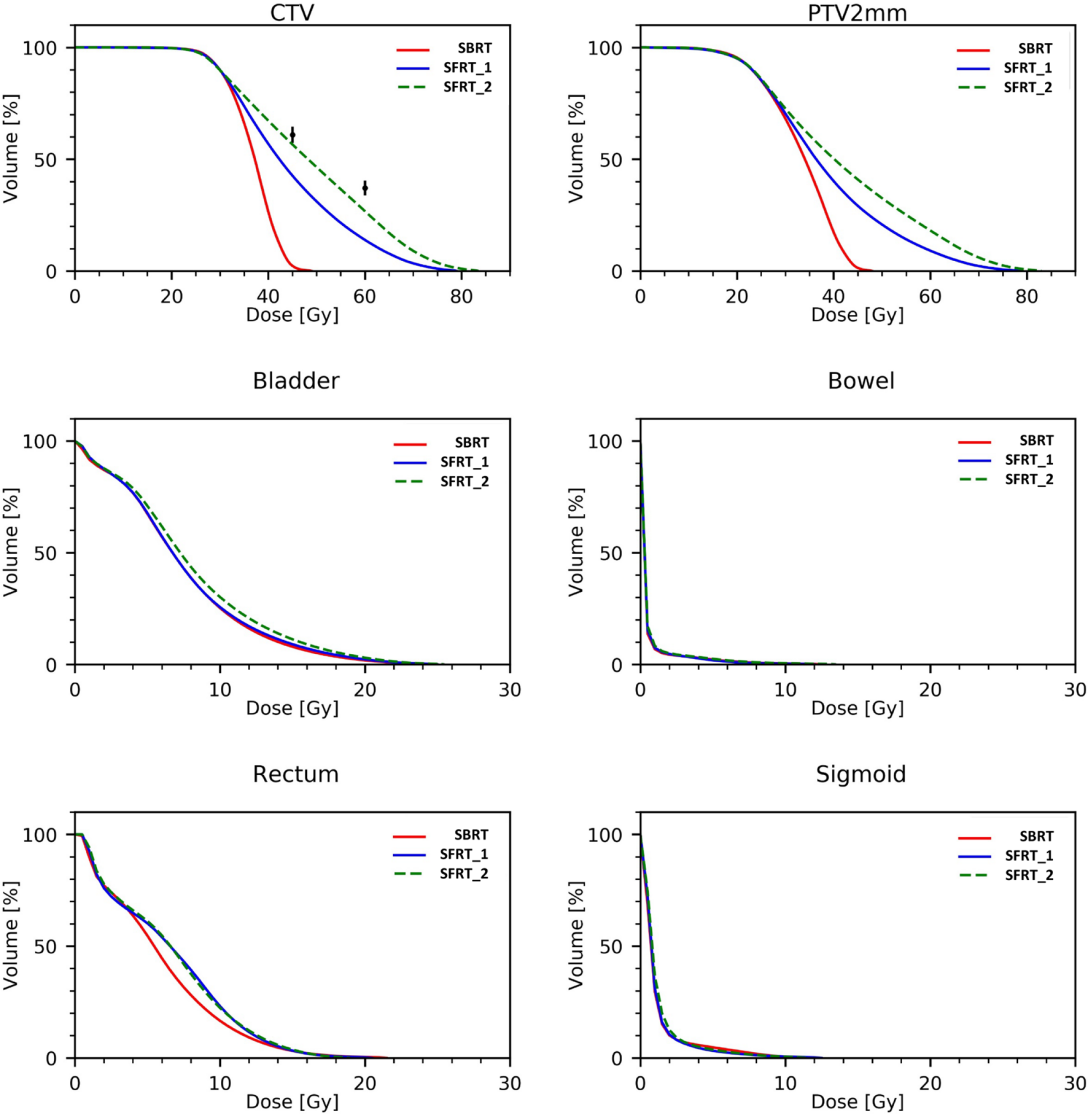
The average dose-volume histograms of the CTV, PTV2mm and the various OARs for the SBRT (solid red line), SFRT_1 (solid blue line) and SFRT_2 (green dashed line) techniques. SBRT is relative to a simple VMAT plan mimicking a stereotactic body radiotherapy approach (SBRT); SFRT_1 and SFRT_2 correspond to the peak-and-valley and peak-only strategies

Figure 3 shows the average DVH for the CTV and the OARs for the three sets of plans; the reference brachytherapy data points for V45 and V60Gy are shown for reference in the CTV plots. It is qualitatively noticeable how all three techniques resulted in equivalent profiles for the OARs while strongly differentiated for the CTV. In Fig. 3, the DVH of the hypothetical PTV defined with 2 mm expansion is also shown.

Additional file 1: Figure 1compl in the complementary materials available in electronic format only, the average DVH for the CTV is shown for SFRT_1 and SFRT_2 with the 1 and 1.95 standard deviations uncertainty bands shown as dashed lines. There is improvement achieved by relaxing the valley constraints (SFRT_2 plans). In this case, the reference brachytherapy data points fall within or nearby the one standard deviation and well within the 1.95 bands.

Table 1 presents the numerical summary of the DVH analysis for the CTV and the OARs. It also includes the coverage values for the virtual 2 and 5 mm expansions of the CTV. Concerning the CTV, while no attempt to generate heterogeneous doses was made in the SBRT plans, the SFRT datasets, generated with the straight rods, demonstrate the possibility of adequately meeting (or approach) the dose-volume metrics used as comparators to brachytherapy. Given the opportunity to personalise the position and length of the rods, this did not compromise the possibility to fulfil the dose-volume constraints to the surrounding OARs. In fact, none of those was on average violated for the bladder, the rectum, the sigmoid and the bowels.

**Table 1:** Summary of the quantitative analysis of the dose volume histograms for the target volume (CTV) and the organs at risk. Included also the data relative to the coverage of the virtual target expansions of 2 and 5 mm. In brackets the upper limit of the constraints. The comparator data derives from brachytherapy treatments. In the objective’s column, the values in brackets correspond to the upper hard limit for the corresponding metrics. The ranges are reported in square brackets

Structure	Parameter	Objective	SFRT_1	SFRT_2	SBRT	Comparator
CTV	D_90%_	30 Gy	30	30	30	–
	V_45Gy_ (150%)	≥ 50–55%	43.1 ± 7.5 [33.0,55.9]	56.6 ± 5.6 [45.4,64.8]	2.3 ± 3.8 [0,10.6]	61.0 ± 3.8 [56.0,66.4]
	V_60Gy_ (200%)	≥ 30%	15.4 ± 5.6 [6.9,23.9]	26.8 ± 6.6 [17.3,34.2]	0 [0,0]	37.2 ± 3.6 [31.6,41.8]
PTV2mm	D_90%_	30 Gy	23.2 ± 1.3 [21.0,24.5]	23.4 ± 1.7 [21.1,24.7]	23.1 ± 1.1 [21.5,24.3]	–
PTV5mm	D_90%_	30 Gy	20.0 ± 1.4 [19.0,21.8]	22.6 ± 2.1 [17.4,22.2]	19.9 ± 1.1 [17.9,26.2]	–
Bladder	D_2cm3_	≤ 23.75 (27.5) Gy	22.4 ± 2.0 [20.2,27.0]	22.6 ± 2.1 [20.6,27.3]	21.6 ± 2.3 [17.9,26.2]	–
Rectum	D_2cm3_	≤ 17.0 (19.45) Gy	13.9 ± 2.9 [11.2,19.6]	13.2 ± 3.0 [10.3,18.7]	12.7 ± 3.9 [8.0,21.6]	–
Sigmoid	D_2cm3_	≤ 19.5 (21.65) Gy	2.6 ± 3.1 [0.5,10.4]	2.8 ± 3.0 [0.5,9.9]	2.5 ± 2.7 [0.4,8.1]	–
Bowel	D_2cm3_	≤ 17.0 (19.45) Gy	9.1 ± 5.9 [1.2,17.6]	9.7 ± 7.3 [1.0,18.4]	9.6 ± 6.6 [2.7,18.0]	–

D_x_: dose received by at least X% or Xcm^3^ of the volume, SFRT: spatially fractionated radiation therapy, SBRT: stereotactic body radiation therapy, CTV: clinical target volume, PTV: planning target volume

Table 2 presents the same data corrected to EQD2 and accounting for the uniform delivery of 45 Gy with external beam therapy before the SFRT boost. In this table, the comparator data from brachytherapy patients were also added for the OARs as well as the EQD2 corrected values from the study of Albuquerque [5] from SBRT for further comparison. SFRT allowed a better sparing for the sigmoid and the bowels while staying within dose tolerances for the bladder and the rectum.

**Table 2:** Biologically corrected EQD2 dose with the inclusion of uniform 45 Gy from external beam radiotherapy prior to the SFRT boost. Comparator data derives from brachytherapy treatments while the last column reports the data from the SBRT study of Albuquerque et al. [5]. The ranges are reported in square brackets

Structure	Param	Obj	SFRT_1	SFRT_2	SBRT	Comparator	Albuquerque
CTV	D90%	84.25 Gy	84.25	84.25	84.25	88.2 ± 1.8	–
	V45Gy (150%)	≥ 50–55%	43.1 ± 7.5 [33.0,55.9]	56.6 ± 5.6 [45.4,64.8]	2.3 ± 3.8 [0,10.6]	61.0 ± 3.8 [56.0,66.4]	–
	V60Gy (200%)	≥ 30%	15.4 ± 5.6 [6.9,23.9]	26.8 ± 6.6 [17.3,34.2]	0 [0,0]	37.2 ± 3.6 [31.6,41.8]	–
PTV2mm	D90%	84.25 GY	72.6 ± 2.2 [69.2,74.7]	72.9 ± 2.2 [72.1,75.3]	72.4 ± 2.1 [69.9,74.4]	–	–
PTV5mm	D90%	84.25 Gy	67.6 ± 2.1 [64.5,70.3]	68.2 ± 2.5 [63.8,71.0]	67.4 ± 2.1 [64.2,69.3]	–	–
Bladder	D_2cm3_	≤ 80.0 Gy	76.6 ± 4.9 [71.7,88.5]	76.9 ± 5.2 [72.6,89.3]	75.0 ± 5.5 [66.7,86.4]	66.5 ± 5.9 [56.8,86.9]	99.6 (*)
Rectum	D_2cm3_	≤ 65.0 Gy	59.5 ± 5.5 [53.5,72.6]	58.0 ± 5.0 [53.7,68.4]	57.8 ± 7.2 [50.6,74.9]	53.1 ± 4.1 [48.0,58.9]	90.6 (*)
Sigmoid	D_2cm3_	≤ 70.0 Gy	45.4 ± 3.2 [44.0,53.7]	45.4 ± 3.1 [43.5,53.1]	45.2 ± 2.5 [43.4,50.6]	61.5 ± 2.7 [57.0,66.2]	80.7 (*)
Bowel	D_2cm3_	≤ 65.0 Gy	52.8 ± 7.7 [43.9,66.1]	52.9 ± 8.4 [45.0,67.8]	54.1 ± 9.1 [45.1,67.0]	54.3 ± 7.1 [46.3,67.3]	67.9 (*)

D_x_: dose received by at least X% or Xcm^3^ of the volume, SFRT: spatially fractionated radiation therapy, SBRT: stereotactic body radiation therapy, CTV: clinical target volume, PTV: planning target volume, (*) median values

#### Target coverage and mitigation strategy

the data shown in Table 1 suggest that with the dose normalisation set to the CTV 90% coverage, the dose fall-off required to guarantee the protection of the OARs would impact the coverage of the hypothetical PTV expansions of 2 and 5 mm. The D_90%_ would drop from an aim of 30 Gy to about 23 Gy for PTV2mm and about 20 Gy for PTV5mm.

Different strategies were considered to mitigate the risk of under-dosage in case of uncertainties in the positioning or organs motion. Table 3 reports the results from the two strategies applied compared against the baseline with simple normalisation at D_90%_ = 30 Gy for the CTV. For simplicity, the data are reported for the peak-and-valley plans only; similar results were achieved for the peak-only dataset.

**Table 3:** The summary of the coverage study. SFRT_1 A: dose normalisation to the maximum dose tolerable for PTV2mm (maximum D90%). SFRT_1 B: plan reoptimized and normalised as in A. In both A and B subset the maximum D90% was determined as the value preventing any constraint violation in the OARs. In the objective’s column, the values in brackets correspond to the upper har limit for the corresponding metrics. The ranges are reported in square brackets

Structure	Parameter	Objective	SFRT_1	SFRT_1 A	SFRT_1 B
CTV	D90%	30 Gy	30	35.3 ± 3.2 [30.3,38.2]	33.1 ± 2.5 [30.1,37.9]
	V45Gy	≥ 50–55%	43.1 ± 7.5 [33.0,55.9]	61.3 ± 13.9 [35.3,77.4]	51.6 ± 13.8 [36.4,76.4]
	V60Gy	≥ 30%	15.4 ± 5.6 [6.9,23.9]	29.2 ± 13.0 [7.2,47.1]	18.7 ± 11.0 [5.4,43.4]
PTV2mm	D90%	30 Gy	23.2 ± 1.3 [21.0,24.5]	27.2 ± 2.8 [22.3,30.6]	28.2 ± 1.8 [25.1,]
PTV5mm	D90%	30 Gy	20.0 ± 1.4 [19.0,21.8]	23.5 ± 2.8 [20.1,27.4]	25.1 ± 1.6 [22.1,26.8]
Bladder	D_2cm3_	< 23.75 (27.5) Gy	22.4 ± 2.0 [20.2,27.0]	26.2 ± 1.7 [23.0,27.4]	26.1 ± 1.3 [24.2,27.4]
Rectum	D_2cm3_	< 17.0 (19.45) Gy	13.9 ± 2.9 [11.2,19.6]	16.2 ± 2.9 [10.4,20.6]	18.6 ± 0.8 [16.9,19.4]
Sigmoid	D_2cm3_	< 19.5 (21.65) Gy	2.6 ± 3.1 [0.5,10.4]	3.0 ± 3.1 [0.6,10.5]	5.3 ± 6.3 [0.6,18.7]
Bowel	D_2cm3_	< 17.0 (19.45) Gy	9.1 ± 5.9 [1.2,17.6]	10.5 ± 6.8 [1.5,19.9]	10.2 ± 6.2 [1.4,17.2]

D_x_: dose received by at least X% or Xcm^3^ of the volume, SFRT: spatially fractionated radiation therapy, SBRT: stereotactic body radiation therapy, CTV: clinical target volume, PTV: planning target volume

The SFRT A and B strategies were similar in terms of the potential mitigation impact on the coverage issue for PTV2mm, raising its D_90%_ from about 23 Gy to about 27–28 Gy (i.e. less than 10% below the ideal value of 30%). D_90%,_ as reported for the PTV5mm, increased by 17.5% (SFRT A) and by 25.5% (SFRT B). As per design, no violations were observed for the OARs, and the final average values for D_2cm3_ were all within the maximum tolerable thresholds for the bladder and rectum. The coverage for the CTV increased, and D90% raised from the fixed 30 Gy of the original plans to 33–35 Gy, i.e. 10–15% higher than the nominal prescription. Consequently, this impacted the V45Gy and V60Gy metrics, which respected the planning aims in the case of SFRT_1 A (the most straightforward approach).

#### The case for bending rods

The use of bending rods was tested in a subset of patients as a complimentary feasibility investigation. Additional file 1: Figure 2compl illustrates a single rod inside the corresponding CTV in 3D view (panel A). The isodose distribution in the colour wash (from 25 to 70 Gy to saturate the display) suggests how the structure can be shaped to follow the patient’s anatomy and keep an ideal separation from the surrounding OARs (panel B). The dose-volume histograms shown in panel C demonstrate the possibility of respecting all the planning aims for the OARs and consolidating the achievement of the V_45Gy_ goal (with the same discrepancy observed for the straight rods relatively to V_60Gy_). The detailed results are not reported but confirm the findings from the simpler rods.

## Discussion

A novel method for the MLC-based generation of virtual patterns for SFRT was proposed in this study and assessed at the planning level for cervix uteri patients. The primary scope of the research was the development of an easily implementable and versatile tool for the realisation of SFRT as a viable solution when standard BT would not be an option or is not available as a clinical service, or would not be suitable for specific patients. The authors by no means propose it as a replacement for standard 3D image-guided intracavitary or intracavitary-interstitial brachytherapy. The advantages of 3D image-based brachytherapy are not only limited to dose distribution, including central hot spots and rapid dose fall-off, but also in the delivery of treatment with no setup margin. This has resulted in excellent outcomes, as seen in a recently published EMBRACE 1 study with 5-year local control of 92% (95% CI 90–93) and low grade 3 and above morbidities [17].

For the present study, we selected patients with HRCTV > 30 cm^3^ to represent patients with residual disease at brachytherapy. This decision was also made to ensure that targets are sufficiently large such that impact of these techniques on OAR can also be studied, especially with large residual tumours after chemoradiation. The planning study was performed using a specific delivery platform (the Halcyon). Still, there are no restrictions in the script or the delivery technique preventing using this SFRT approach on other platforms (any C-Arm linac) capable of operating VMAT treatments. The proposed software tools allow for the definition of virtual rods within any given target volume. These rods, of variable diameter, inter-centre separation and length could be defined as straight patterns (oriented at any angle in the XZ and YZ planes) or individualised with a carving following individual patients’ anatomy.

The study constitutes a proof of principle of the technique’s feasibility, and the selection of 10 cases from an earlier investigation [15] allowed to investigate its potential in a realistic clinical setting. The sample size would be insufficient in any clinical trial. Still, it appears to be sufficient and consistent with state-of-the-art practices in treatment planning studies for a dosimetric proof of concept.

The results showed that some dose-volume metrics (V_45Gy_ and V_60Gy_) used as a comparator with brachytherapy could be replicated with the virtualised approach through the rod technique. This may lead to a local control outcome comparable to BT. Secondly, all the OAR dose-volume constraints (derived from BT standards) could be respected. The EQD2 corrected total near-to-maximum doses (D_2cm3_) for the OARs from the present study (Table 2) are largely lower than the corresponding data from the Albuquerque [5] study (median of 90.6, 80.7, 67.9 and 99.6 Gy for the rectum, sigmoid, small bowel and bladder respectively) and consistent with the brachytherapy data used as a comparator. One feature related to the use of external radiotherapy is the presence of a dose bath (shown with the 5 Gy lower threshold of the colourwash in Fig. 2) which would be, of course, not present (or much reduced) with conventional brachytherapy. Nevertheless, the current investigation does not aim to provide a one-to-one replacement of brachytherapy but rather a solution where BT is not viable; therefore, different dose patterns are expected and considered clinically.

As a limit of the study, the comparison was performed only agains two dose-volume metrics and not based on DVHs and dose distributions from brachytherapy plans. The main reason for this was the un-availability of CT image datasets of the same patients with and without brachytherapy implant in place.

One relevant aspect of any external beam treatment technique is the robustness (or lack thereof) with respect to position and organ’s motion uncertainties. As a first-order assessment of the quality of the proposed solution, the target volume was expanded by 2 and 5 mm (PTV expansions not primarily considered in the optimisation). The data showed a decent coverage of these expansions but with some significant reduction (D_90%_ for PTV2mm was of ~ 23 Gy without any mitigation strategy). If for clinical reasons, better coverage of this PTV expansion would be desirable (to make plans more robust), we proposed alternative strategies with renormalisation and eventually re-planning incorporating the PTV in the process. The results demonstrated coverage of the PTV 2 mm approaching the ideal level of 30 Gy for D_90%_ (with a deviation of less than 10%). As a result, the dose to the CTV was increased by 10–15% (with a benefit for V_45Gy_ and V_60Gy_) while none of the absolute upper dose-volume constraints for the OARs was violated (particularly for the bladder and the rectum).

Dosimetric validation of the proposed technique with an appropriate set of measurements will be part of a follow-up investigation. If results are consistent and acceptable, a phase I trial could be considered in subgroups of cervical cancer patients. These might include patients with post-radiation recurrences that are not amenable to surgical or brachytherapy salvage. The presence of gross residual disease after external radiation precludes brachytherapy or unfavourable anatomy leads to suboptimal brachytherapy target coverage. In addition, patients with large volume recurrences of endometrial, vaginal cancers and pelvic sarcomas that are not amenable for salvage surgical resection or salvage radiation ± brachytherapy could be considered. Furthermore, there may be medical contraindication to any anaesthesia procedures and, finally, the patient’s choice to refuse brachytherapy treatments.

The current study has obvious limitations. First is the selection of the target volume. In clinical practice, patients would ideally receive dedicated imaging for brachytherapy when nearing the end of the conventional external beam course. This would reduce tumour volumes compared to the initial planning imaging used for the present in-silico study. This aspect should be carefully addressed in the design and execution of a phase I trial. Still, it has a limited impact in terms of the current dosimetric in-silico assessment of the technique. The caveat is that with smaller volumes, a different set of geometrical patterns might be needed (particularly with respect to the diameter and separation of the rods) and possibly in terms of their curvature. To confirm this, it is worth mentioning that the volume of the targets from our study was relatively larger than the comparator BT volumes (48.3 ± 22.4 versus 29.2 ± 9.6 cm^3^). This fact justifies the hypothesis that the proposed technique could also apply to patients poorly responding to EBRT and may represent volumes of recurrent diseases where BT may be technically more challenging.

As a second consideration, the positional variation of target and organs at risk, which could be either interfraction or intrafraction, might be significant. Therefore, appropriate image guidance protocols should be applied before treatment. Also, uncertainty mitigation methods should be applied in the planning process. The data shown in this study suggest that in the range of 2–5 mm, sufficient target coverage might be granted. A more sophisticated approach might require daily re-planning/re-optimisation if the local resources would allow for it.

Another area of future investigation should be the actual deliverability of the treatment plans. Dedicated pre-treatment quality assurance procedures and protocols should be developed and validated, especially for inter-fraction and intra-fraction setup uncertainties and organ motion. Firstly, the planning method proposed is based on clinically released and utilised optimisation engines, routinely used for the delivery of SBRT of small and multiple tumours (e.g. multiple brain metastases) [18], and with very high doses per fraction [19]. Secondly, the size of the virtual rods (5 mm) and the inter-centre separations (15 mm) correspond to the resolution of the MLC for the rods and to three times it for the separation (leaving an “empty” space of 10 mm, i.e. the equivalent of 2 MLC leaves between the rod edges). These values appear to be properly compatible with the dose calculation resolution of 2.5 mm chosen (which could be further improved up to 1 mm). On the contrary, very narrow apertures (physical or virtual) might incur relevant dosimetric uncertainties proper to very small field dosimetry; this fact is not of concern with the proposed solution. Besides, the flexibility of the novel tool presented here should easily enable the adaptation of SFRT to larger or smaller targets.

In the present report, bending/carving virtual rods were qualitatively presented in the Additional file 1: complementary materials to enlighten the potential flexibility of the approach. It is obvious that more extensive dosimetric validation should be performed, although the possibility to further personalise the SFRT patterns appears to be a relevant advantage if actual clinical trials are designed and implemented. Again, the proposed environment would enable clinicians to select either straight or carving virtual rods at their preference.

The application domain of the novel method for SFRT proposed in this study is not limited to gynecologic cancer boost treatment. Still, it might be applied to other indications with eventually bulkier tumour volumes in different anatomical districts. Further dose planning investigation should confirm the assumption, but the technique has not a priori limits in this respect.

## Conclusion

A novel method for the generation of arbitrary spatially fractionated patterns was proposed and investigated at the treatment planning level for delivering the boost component of a radiotherapy course in cervical cancer patients. Based on a sample of 10 patients, the dosimetric results are promising and allowed to comply with dose-volume constraints on the organs at risk and provide adequate irradiation of the target volume, which are closer to brachytherapy dose profiles than IMRT and SBR techniques. Further investigations on dosimetric deliverability and clinical feasibility with a phase I trial could be considered further steps.

## Supplementary Information

**Additional file 1**. Complementary materials on DVH statistical uncertainty for the peak-only and peak-and-valley approach and on the bending rods method.

## Data Availability

The datasets used and analysed during the current study are available from the corresponding author.

## Abbreviations

BT: Brachytherapy
CT: Computed tomography
CTV: Clinical target volume
DICOM: Digital Imaging and Communications in Medicine
DVH: Dose Volume histograms
EQD2: Equivalent dose for 2 Gy fractions
Gy: Gray
LMIC: Low-mid income countries
MLC: Multileaf collimators
MV: Megavolt
OAR: Organ at risk
PTV: Planning target volume
SABR: Stereotactic ablative radiation therapy
SBRT: Stereotactic body radiation therapy
SFRT: Spatially fractionated radiation therapy
VMAT: Volumetric modulated arc therapy
