# Supplementary material for: A novel external beam radiotherapy method for cervical cancer patients using virtual straight or bending boost areas; an in-silico feasibility study
Source: Radiat Oncol. 2021 Jun 14;16:110. doi: 10.1186/s13014-021-01838-x (PMC8201836; doi:10.1186/s13014-021-01838-x)
Supplement: Supplementary file 1 — Additional file 1. Complementary materials on DVH statistical uncertainty for the peak-only and peak-and-valley approach and on the bending rods method. [file 13014_2021_1838_MOESM1_ESM.docx]

**A novel external beam radiotherapy method for cervical cancer patients using virtual straight or bending boost areas; an in-silico feasibility study.**

**Complementary materials**

**Figure 1compl: the average DVH for CTV in the SFRT_1 and SFRT_2 cases with the 1 and 1.95 standard deviations uncertainty bands.**


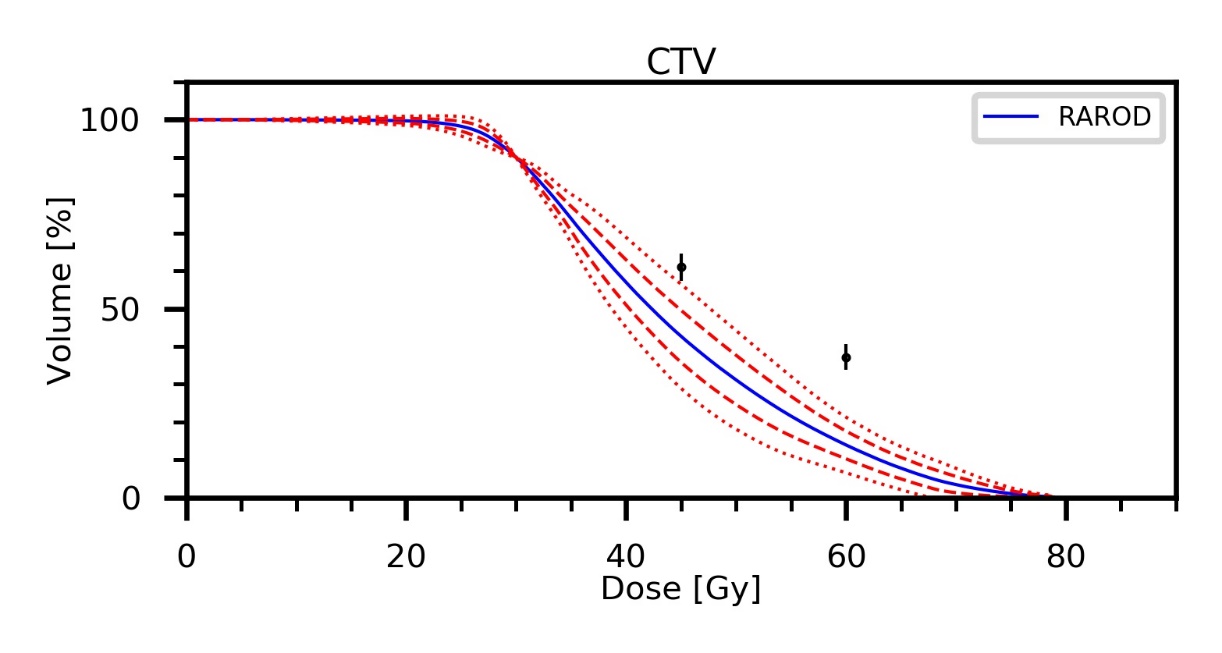


peak-and-valley

SFRT_1


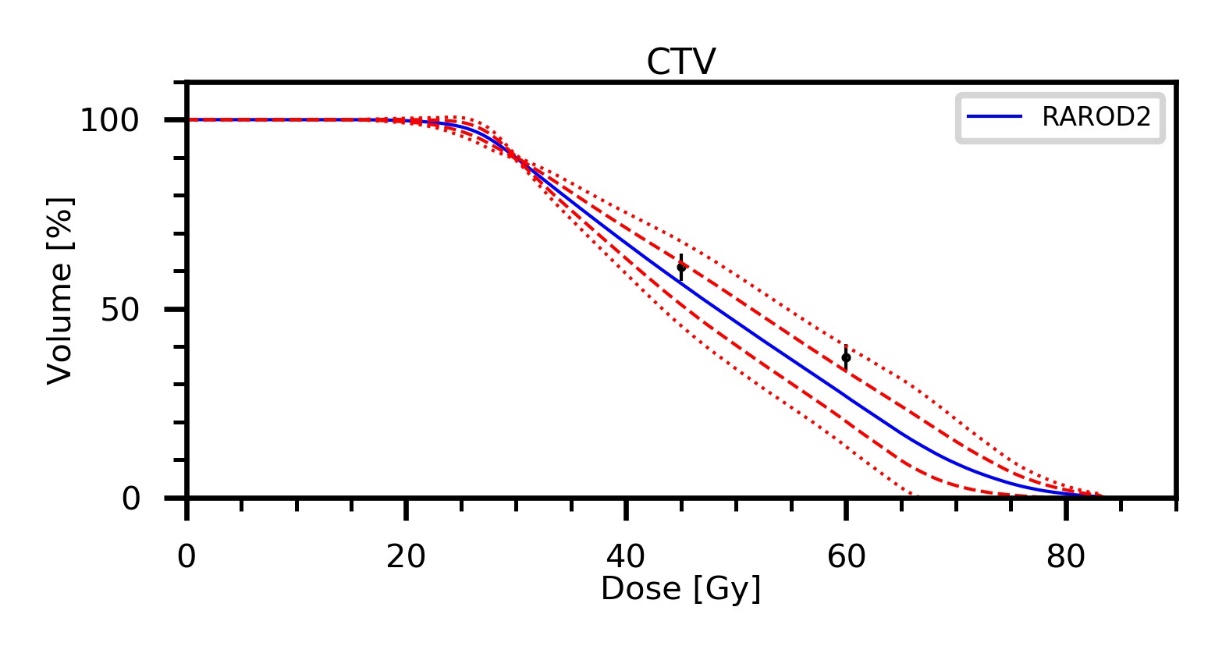


peak-only

SFRT_2

.

**Figure 2Compl:** An example case of a bending rod in 3D view and with isodose colour-wash. A: three dimensional view of the CTV and the bending rod (range) within its volume. B: the dose distribution in color wash for a reconstructed plan. The overlays show the bladder, the CTV and the bending rod contours. C) The dose-volume histogram is relative to the CTV and the various OARS. The points on the DVH are the planning aims for each structure. CTV: blue; Bladder: cyan; rectum: orange; sigmoid: yellow; bowels: brown


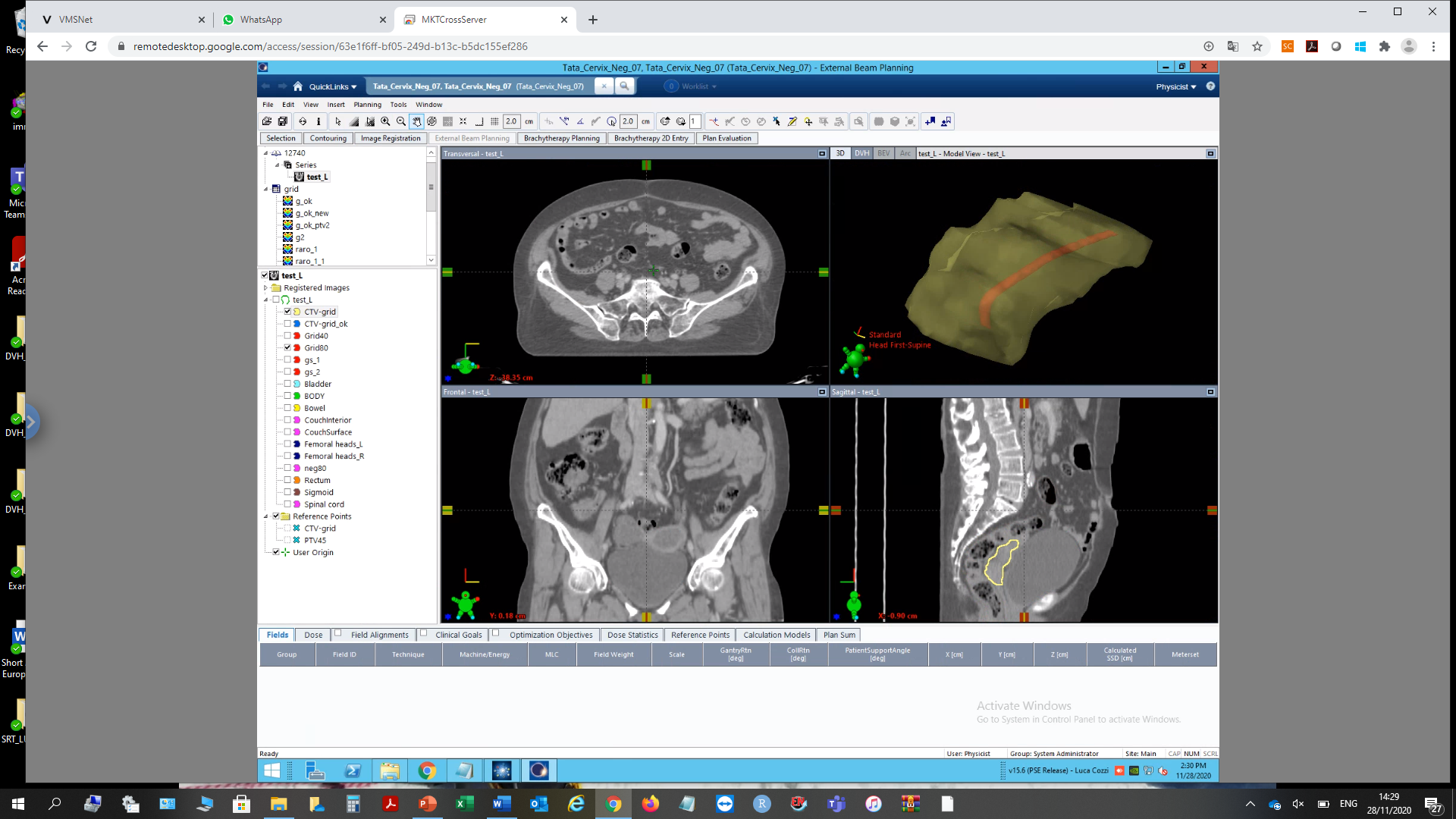

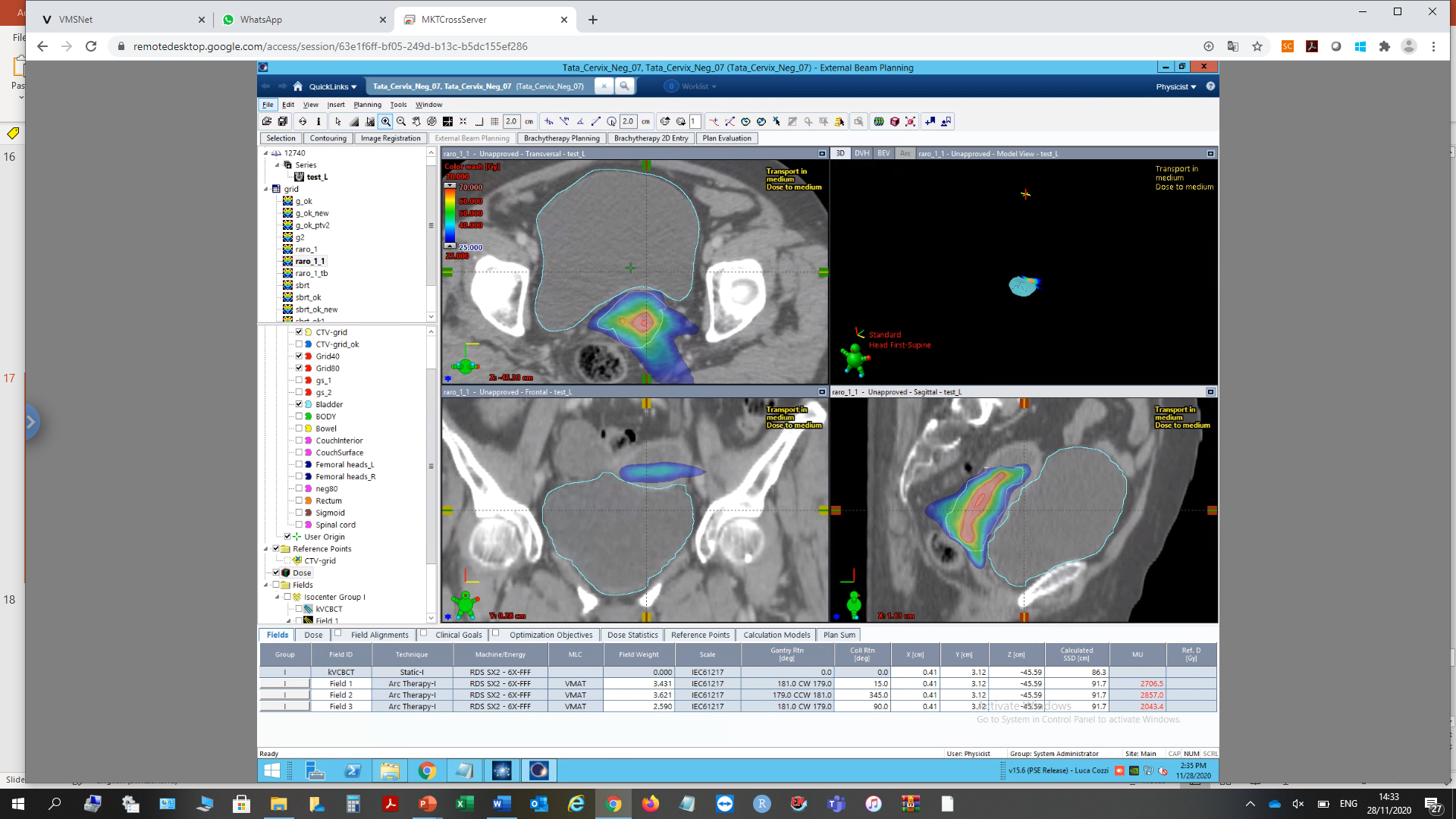

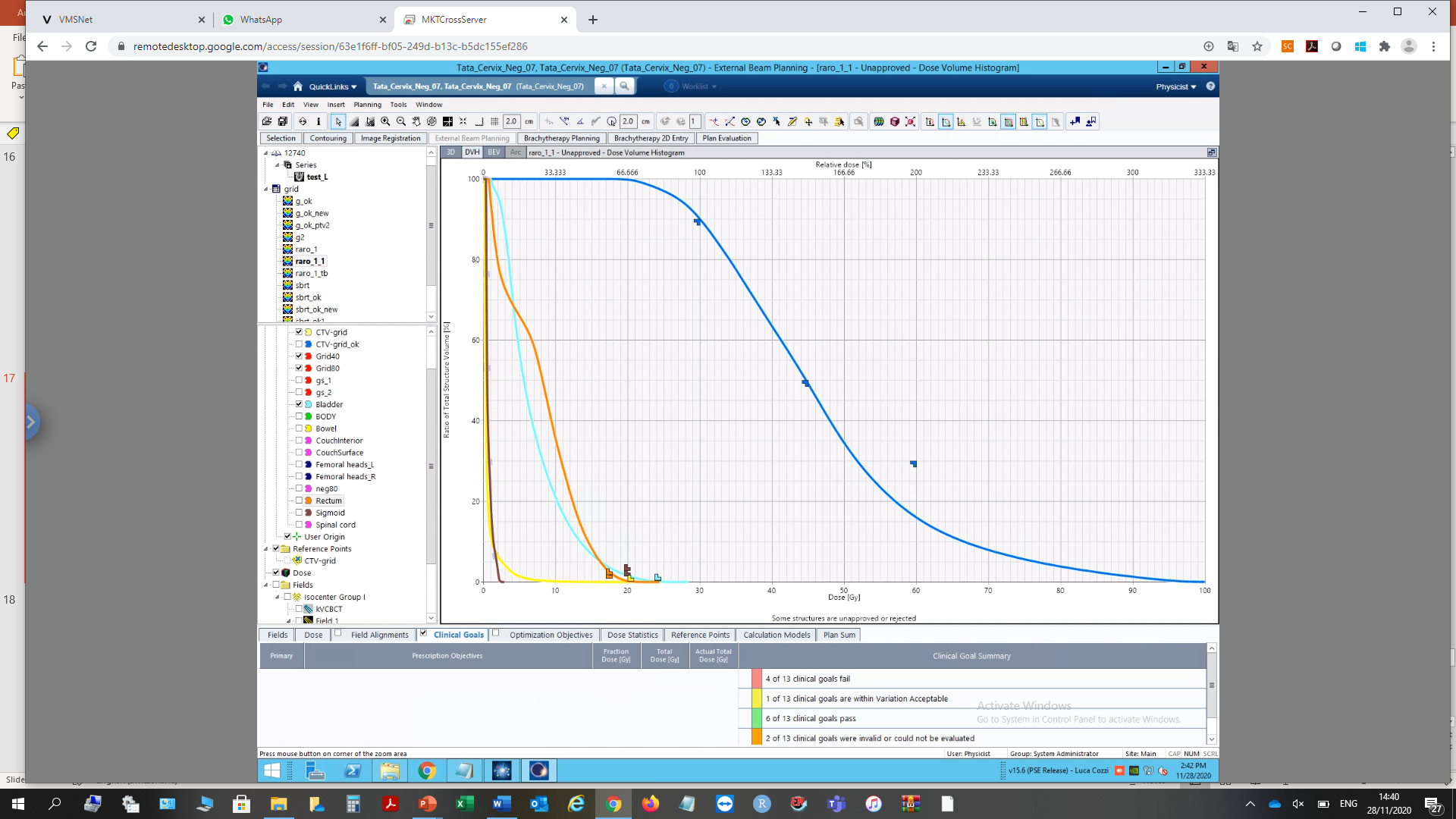


100

80

60

40

20

0

0 10 20 30 40 50 60 70 80 90 100

Dose [Gy]

Volume [%]

A

B

C
